# Using allele scores to identify confounding by reverse causation: studies of alcohol consumption as an exemplar

**DOI:** 10.1093/ije/dyac165

**Published:** 2022-08-18

**Authors:** Hannah M Sallis, Tom Palmer, Kate Tilling, George Davey Smith, Marcus R Munafò

**Affiliations:** MRC Integrative Epidemiology Unit at the University of Bristol, Bristol, UK; Population Health Sciences, Bristol Medical School, University of Bristol, Bristol, UK; Centre for Academic Mental Health, Population Health Sciences, Bristol Medical School, University of Bristol, Bristol, UK; School of Psychological Science, University of Bristol, Bristol, UK; MRC Integrative Epidemiology Unit at the University of Bristol, Bristol, UK; Population Health Sciences, Bristol Medical School, University of Bristol, Bristol, UK; MRC Integrative Epidemiology Unit at the University of Bristol, Bristol, UK; Population Health Sciences, Bristol Medical School, University of Bristol, Bristol, UK; MRC Integrative Epidemiology Unit at the University of Bristol, Bristol, UK; Population Health Sciences, Bristol Medical School, University of Bristol, Bristol, UK; MRC Integrative Epidemiology Unit at the University of Bristol, Bristol, UK; School of Psychological Science, University of Bristol, Bristol, UK; NIHR Biomedical Research Centre, University Hospitals Bristol NHS Foundation Trust and University of Bristol, Bristol, UK

**Keywords:** Reverse causation, confounding, Mendelian randomization, allele scores

## Abstract

**Background:**

Mendelian randomization (MR) is a form of instrumental variable analysis used to investigate causality using observational data. Another important, although less frequently applied, use of this technique is to investigate confounding due to reverse causality.

**Methods:**

We used a form of reverse MR and data from UK Biobank in a proof-of-principle study to investigate confounding due to reverse causation. Here we focus on the association between alcohol consumption (exposure) and outcomes including educational attainment, and physical and mental health. First, we examined the observational relationship between alcohol consumption and these outcomes. Allele scores were then derived for educational attainment, and physical and mental health, and the association with alcohol consumption (as the outcome) was explored. Sample sizes ranged from 114 941–336 473 in observational analyses and 142 093–336 818 in genetic analyses.

**Results:**

Conventional observational analyses indicated associations between alcohol consumption and a number of outcomes (e.g. neuroticism, body mass index, educational attainment). Analyses using allele scores suggested evidence of reverse causation for several of these relationships (in particular physical health and educational attainment).

**Conclusion:**

Allele scores allow us to investigate reverse causation in observational studies. Our findings suggest that observed associations implying beneficial effects of alcohol consumption may be due to confounding by reverse causation in many cases.

Key Messages

- Findings from conventional observational studies may be distorted by reverse causation.
- Reverse Mendelian randomization (MR) can be used to directly test reverse causation in observational epidemiological studies where bidirectional MR is not possible.
- Future extensions to this method could enable us to adjust observational estimates for reverse causation.
- Our findings suggest that observed associations suggesting beneficial effects of alcohol consumption may be largely due to confounding by reverse causation.

## Introduction

Mendelian randomization (MR) is often performed in the conventional direction to investigate causal effects of an exposure (e.g. alcohol consumption) on an outcome (e.g. mental health). However, it can also be used in the opposite direction to explore reverse causality. Since genetic variants are randomly and independently assigned at conception, we can use these as unconfounded proxies for our exposure that are not subject to reverse causality.^1^^,^^2^ If reverse causality is causing observed associations, genetic variants associated with the outcome would be associated with the ‘exposure’. However, if the observed association between the exposure and the outcome was confounded (e.g. by socio-demographic characteristics),^3^ we should not see an association between the genetic variants for the outcome and the exposure, although this is contingent on the exposure being correctly specified, since genetic variants associated with socio-demographic characteristics would be associated with both the exposure and outcome. Therefore it is important that we minimize this form of pleiotropy by restricting our instruments to variants that could plausibly act on our exposure.^4^ Our approach is a form of bidirectional MR analysis.^5^ Bidirectional MR is typically performed when there is a plausible causal pathway acting in both directions, but MR in the reverse direction is rarely used to directly investigate confounding by reverse causality. Here we use allele scores (based on genome-wide significant hits) for the outcomes of interest to help us to investigate confounding due to reverse causation in observational studies.

In our proof-of-principle example, we focus on alcohol consumption and educational attainment, and mental and physical health outcomes; therefore in this case it would have been possible to perform a complete bidirectional MR as genetic instruments are available for alcohol consumption. However, our aim was to illustrate that this form of MR enables us to look at reverse causation and this method could therefore be extended to look at exposures that are either difficult to instrument (e.g. cycling) or where the instrument itself may be problematic due to issues with pleiotropy or heterogeneity (e.g. smoking initiation, where it is unclear whether the genetic instrument acts directly via biological pathways or indirectly through behavioural pathways^6^).

**Figure 1:**
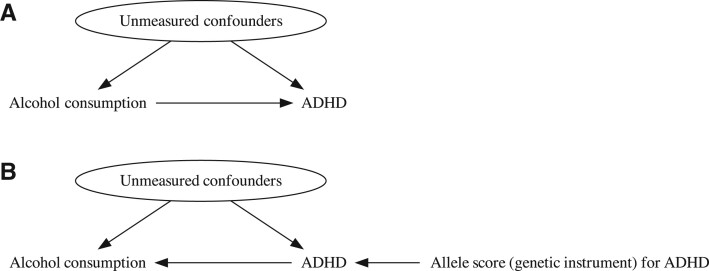
Reverse Mendelian randomization Directed acyclic graphs (DAGs) for our models. (a) We estimate the association between alcohol consumption on one of our mental and physical health phenotypes [attention-deficit hyperactivity disorder (ADHD)]. (b) Testing the presence of a causal effect of ADHD on alcohol consumption using the allele score as an instrumental variable for ADHD under the Mendelian randomization approach.

We used data from the UK Biobank (UKB) study^7^ to investigate this phenomenon. Our approach is described in Figure 1. First, we investigated the observational association between alcohol consumption and mental and physical health. An observational association could be due to (i) a true causal effect of alcohol consumption on these outcomes, (ii) confounding due to e.g. social characteristics, (iii) reverse causation (i.e. a causal effect of mental/physical health on alcohol consumption, or the ‘sick quitter’ hypothesis) or (iv) a combination of the above. Second, we derived allele scores (based on single-nucleotide polymorphisms identified in a separate genome-wide association study with genome-wide significance; *P* < 5 × 10^–8^) for each of the mental and physical health outcomes and investigated the association between genetic liability for each outcome and several alcohol consumption phenotypes. Under the three core instrumental variable conditions a test of association between the instrument and outcome is a test of the presence of a causal effect of the exposure on an outcome.^8^ As can be seen from Figure 1, in our conventional observational analysis alcohol consumption was considered as the exposure, whereas in our allele score analysis it is considered as the outcome. Evidence of an association in our allele score analysis would suggest that at least some of the observed relationship is likely due to reverse causality.

## Methods

### UKB study

The UKB study is a large population cohort consisting of ∼500 000 individuals recruited in the UK between 2006 and 2010. Eligible individuals were aged between 40 and 69 years at the time of recruitment and living within 25 miles of an assessment centre (22 assessment centres were based across England, Wales and Scotland). Approximately 9.2 million individuals were invited to take part, with ∼5.5% of these participating in the baseline assessment.^7^

### Measures

#### Educational attainment, and physical and mental health outcomes

Our focus was primarily on phenotypes for which there were both observed measures collected within UKB and genetic variants available to create allele scores (listed and defined in Table 1). We extracted several mental health outcomes including clinician diagnoses of depression, attention-deficit hyperactivity disorder (ADHD), autism spectrum disorder (ASD), schizophrenia, bipolar disorder, anxiety and neuroticism summary scores,^9^ along with measures of body mass index (BMI), height, educational attainment, smoking status and blood pressure.

**Table 1:** Genome-wide association studies used to derive allele scores for each outcome of interest in UK Biobank

Outcome	Study	Number of SNPs in allele score
ADHD	Demontis *et al*.^11^	11
Anxiety	Meier *et al*.^12^	10
Autism spectrum disorder	Grove *et al*.^13^	4
Bipolar disorder	Stahl *et al*.^14^	21
Depression^a^	Wray *et al*.^5^	40
Schizophrenia	Pardinas *et al*.^16^	130
Extraversion	Lo *et al*.^17^	5
Neuroticism	Lo *et al*.^17^	1
BMI	Locke *et al*.^18^	76
Height	Wood *et al*.^19^	694
Education	Okbay *et al*.^20^	72
Smoking initiation^b^	Liu *et al*.^21^	368
SBP	Ehret *et al*.^22^	29
Hypertension	Ehret *et al*.^22^	29

a Discovery sample included the interim release of UKB; these individuals were excluded from analyses using this risk score.

b Discovery sample included UKB; summary statistics excluding UKB were used to derive the allele score.

ADHD, attention-deficit hyperactivity disorder; BMI, body mass index; SBP, systolic blood pressure.

#### Alcohol consumption

Self-reported alcohol consumption was measured at the baseline assessment visit and two follow-up clinics. Participants were asked about (i) current drinking status (‘never’, ‘current’ or ‘former’), (ii) frequency of drinking (‘daily or almost daily’, ‘three or four times a week’, once or twice a week’, ‘one to three times a month’, ‘special occasions only’, ‘never’) and (iii) average weekly or monthly intake of certain types of alcohol (‘beer and cider’, ‘champagne and white wine’, ‘fortified wine’, ‘red wine’, ‘spirits’, ‘other’). Former drinkers were also asked whether they had stopped drinking for medical reasons.

Frequency of drinking and weekly intake were weighted according to alcohol type to derive an estimate of units consumed per week as described in Supplementary materials (available as Supplementary data at *IJE* online).

We derived three distinct phenotypes relating to alcohol consumption:

- drinking status (categorized as two binary variables: ever/never drinker and former/current drinker);
- units per week;
- extreme drinking—a binary variable comparing individuals drinking >50 units per week to the rest of the sample.

#### Covariates

Age, sex, highest educational qualification and total household income were measured at the baseline assessment visit.

#### Allele scores

Genotyped data were available for 336 818 participants in UKB. Details of genotyping and quality control measures are available in the Supplementary materials (available as Supplementary data at *IJE* online). Quality control filtering of the UKB data was conducted by R. Mitchell, G. Hemani, T. Dudding, L. Corbin, S. Harrison, L. Paternoster^10^ as described in the published protocol (doi:10.5523/bris.1ovaau5sxunp2cv8rcy88688v).

Allele scores were derived for a number of psychiatric, personality, physical health and education phenotypes.^11–22^ These were based on genome-wide hits (*P* < 5 × 10^–8^) from a recent genome-wide association study (GWAS) of each outcome that did not include UKB in the discovery sample (see Table 1 for full details). We included single nucleotide polymorphisms (SNPs) reported as independent in the original GWAS publications. For each phenotype, a weighted allele score was created for each participant in UKB using plink v1.90^23^ and standardized prior to analysis. Effect estimates from the discovery GWAS (as described in Table 1) were used to weight the scores.

### Statistical analysis

#### Phenotypic analyses

Logistic/linear regression models were used to investigate the association between alcohol consumption (as an exposure) and measures of physical and mental health (including psychiatric and personality traits, physical health) and educational attainment. The alcohol consumption phenotypes were drinking status (ever/never and former/current drinker), alcohol consumption (weekly units—continuous and categorical) and extreme weekly drinking. All models were adjusted for sex, age at clinic and a measure of socio-economic position (where education was the outcome we adjusted for total household income; for all other analyses we adjusted for highest qualification).

#### Allele score analyses

Allele scores were derived as described above (see Table 1 for a complete list). Logistic/linear regression analyses were performed as appropriate to explore the association with each alcohol consumption phenotype (as an outcome). All analyses were adjusted for sex and the first 10 principal components of ancestry. When using the depression scores, participants in the interim release of UKB genetic data were excluded as they were included in the discovery sample for this GWAS.^15^ All analyses were restricted to individuals of self-reported White European ancestry with alcohol consumption and genetic data available.

#### Sensitivity analyses

Analyses were first performed on the whole sample for whom data on relevant phenotype, alcohol and genetic data were available. As a sensitivity analysis, individuals reporting weekly alcohol intake of >100 units were excluded from the main analysis of units of alcohol consumption in an attempt to remove outliers and improve the normality of the distribution. The models were re-run including all individuals with data on estimated weekly alcohol intake to investigate the effect of these outliers on the estimates.

## Results

### Phenotypic analyses

#### Drinking status

**Figure 2:**
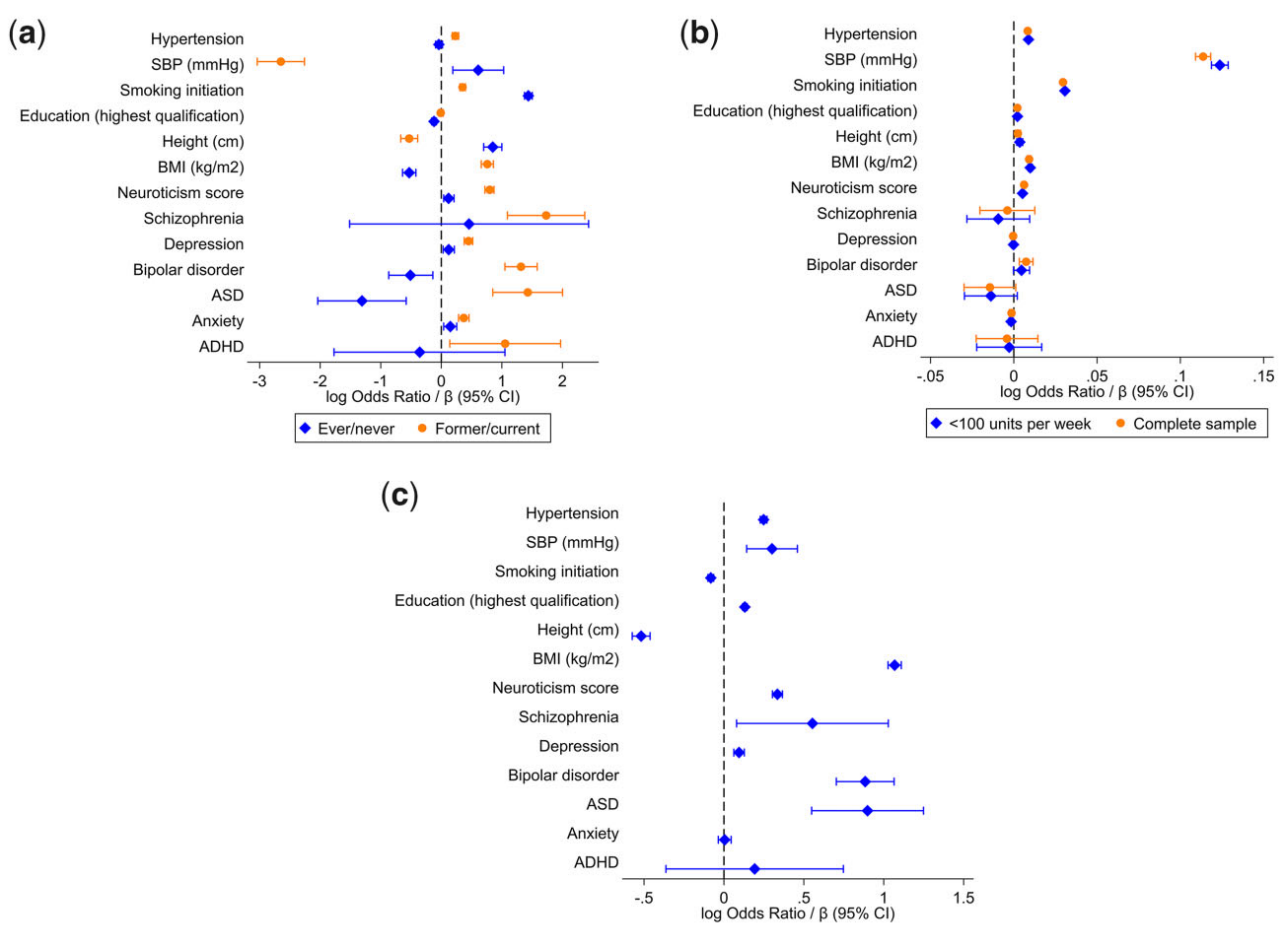
Results from phenotypic analysis of alcohol consumption (exposure) with measures of educational attainment, physical and mental health Alcohol consumption is measured as (a) drinking status (ever compared with never*, and among ‘ever’ drinkers, former compared with current**), (b) weekly alcohol consumption (units per week, in those drinking <100 units per week only and in the whole sample), (c) extreme drinking (individuals consuming >50 units per week compared with the rest of the sample***). Reference groups are as follows: *never drinkers; **current drinkers, ***≤50 units per week. SBP, systolic blood pressure; BMI, body mass index; ASD, autism spectrum disorder; ADHD, attention-deficit hyperactivity disorder.

We found strong evidence of a phenotypic association between ever drinking and increased depression, anxiety, neuroticism, smoking initiation, systolic blood pressure (SBP) and height. Conversely, we found strong evidence of an association between ever drinking and decreased ASD, BMI and education (Figure 2a and Supplementary Table S1, available as Supplementary data at *IJE* online).

When looking among the sample of ‘ever’ drinkers, we found evidence of an association with being a former drinker and increased ADHD, anxiety, ASD, bipolar disorder, depression, schizophrenia, neuroticism, BMI, smoking initiation and hypertension. We found some evidence that being a former drinker was associated with decreased height and SBP (Figure 2a and Supplementary Table S1, available as Supplementary data at *IJE* online).

#### Alcohol consumption

When looking at alcohol consumption as a continuous measure of weekly intake, we found strong evidence of a linear association between weekly units consumed and increased neuroticism, BMI, height, education, smoking initiation, SBP and hypertension (Figure 2b and Supplementary Table S2, available as Supplementary data at *IJE* online). Initially, we restricted to individuals drinking <100 units per week in an attempt to remove outliers; in this instance, we see little evidence of an association between alcohol consumption and bipolar disorder. When these individuals were included, the association between bipolar disorder and increased weekly units became much stronger, although the size of this effect remained small (Figure 2b and Supplementary Table S2, available as Supplementary data at *IJE* online).

#### Extreme drinking

When comparing individuals drinking >50 units per week with the rest of the sample, we found strong evidence of an association with increased ASD, bipolar disorder, depression, schizophrenia, neuroticism, BMI, education, SBP and hypertension. Extreme drinking was also associated with decreased height and smoking initiation (Figure 2c and Supplementary Table S3, available as Supplementary data at *IJE* online).

### Allele score analyses

#### Drinking status

**Figure 3:**
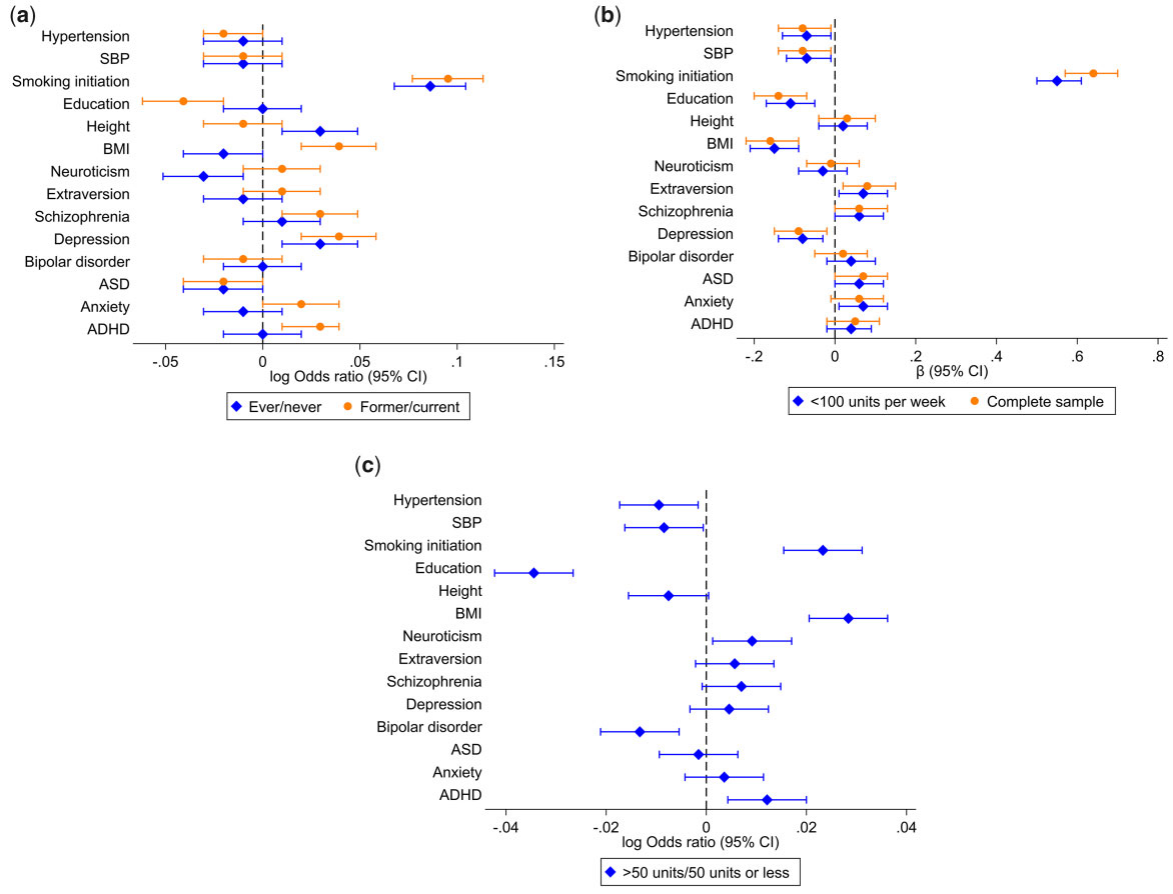
Results from allele score analysis of educational attainment, physical and mental health. (a) Drinking status (ever compared with never*, and among ‘ever’ drinkers, former compared with current**), (b) weekly alcohol consumption (units per week, in those drinking <100 units per week only and in the whole sample), (c) extreme drinking (individuals consuming >50 units per week compared with the rest of the sample***). Reference groups are as follows: *never drinkers, **current drinkers, ***≤50 units per week. SBP, systolic blood pressure; BMI, body mass index; ASD, autism spectrum disorder; ADHD, attention-deficit hyperactivity disorder.

We observed strong evidence of an effect of the allele scores for depression, height and smoking initiation on increased odds of being an ever drinker. The allele scores for neuroticism and BMI were associated with decreased odds of drinking (Figure 3a and Supplementary Table S4, available as Supplementary data at *IJE* online).

When comparing former and current drinkers, we found evidence of an effect of allele scores for ADHD, depression, schizophrenia, BMI and smoking initiation, and increased odds of being a former drinker. Allele scores for ASD and education were associated with lower odds of being a former drinker (Figure 3a and Supplementary Table S4, available as Supplementary data at *IJE* online).

#### Alcohol consumption

When analysing units consumed per week as a continuous measure, we observed evidence of an effect of the allele scores for anxiety, ASD, schizophrenia, extraversion and smoking initiation on increased weekly units of alcohol consumption. Scores for depression, BMI, education, SBP and hypertension were associated with drinking fewer units per week (Figure 3b and Supplementary Table S5, available as Supplementary data at *IJE* online).

#### Extreme drinking

We found evidence of an effect of allele scores for ADHD, neuroticism, BMI and smoking initiation and drinking >50 units per week. Scores for bipolar disorder, education, SBP and hypertension were associated with lower odds of drinking >50 units per week (Figure 3c and Supplementary Table S6, available as Supplementary data at *IJE* online).

## Discussion

We used data from UKB to investigate the role of reverse causation in observational associations using a reverse MR analysis. Reverse causation is a specific form of confounding. Although it is difficult to distinguish between unmeasured confounding and reverse causation in our observational analyses, we can use polygenic scores in a reverse MR approach to help us to investigate reverse causation. We focused on the association between alcohol consumption and health to demonstrate proof of principle. First, we looked at the phenotypic association between alcohol consumption and a number of physical and mental health outcomes and educational attainment. We then investigated the association between genetic liability for these outcomes with several alcohol consumption phenotypes to test whether physical and mental health and educational attainment might be influencing alcohol consumption. Nearly all our conventional observational analyses suggested strong evidence of an association between alcohol use and physical and mental health, which might be taken to imply a causal effect of alcohol use on these outcomes. However, analysis using allele scores to investigate these associations in the reverse direction provided evidence of reverse causation (i.e. that physical and mental health outcomes do indeed influence alcohol use). Where we found no evidence for an association between allele scores and alcohol consumption, we are unable to determine whether the observed relationships reflect a genuine causal effect of alcohol consumption or are subject to other forms of confounding.

In our example of alcohol and physical and mental health, it would be possible to perform a bidirectional MR as instruments exist for both the exposure and outcomes. We did not perform these analyses here as this was intended to be a proof of principle of using risk scores in the reverse direction, in the case in which the exposure did not have any valid instruments. In many cases, there is unlikely to be a valid (genetic) instrument for the phenotypic exposure. For example, studies have suggested that cycling is protective for cardiovascular disease (CVD).^24^ Although there are genetic instruments available for CVD, there is no genetic instrument for cycling, precluding the use of traditional MR analyses to establish a causal effect of cycling on CVD. In this case, we could use the reverse MR proposed here to investigate whether allele scores for CVD ‘cause’ cycling, which would suggest that any observational associations are subject to reverse causation. This method could be extended to adjust the conventional observational estimates to account for this reverse causation but this is outside the scope of the current study. In this study, individual-level data were available for both phenotype and genotype enabling us to directly compare the strength of evidence from each analysis. However, it may be possible to compare the association between observational data sets without genetic data available and estimates from two sample MR analyses, where the underlying populations being sampled are the same.

Here, we first examined the relationship between alcohol consumption and depression in UKB before moving on to investigate the association between alcohol consumption and other, less well-studied outcomes. Observationally, we found that former drinkers report higher levels of depression, consistently with a protective effect of moderate levels of alcohol consumption. However, an increased genetic liability for depression was associated with greater risk of being a drinker, but also a greater chance of stopping drinking once they had started. This suggests that the relationship is potentially due to the ‘sick quitter’ hypothesis rather than a beneficial effect of alcohol, and that individuals with depression were simply more likely to abstain from drinking. This is in line with work by Polimanti and colleagues which found that major depression is genetically correlated with problematic drinking.^25^

For several of the other outcomes we investigated, we observed a consistent direction of effect between our conventional observational and genetic analyses, although the strength of the observational evidence was greater and effect estimates were larger than those suggested by analysis of the allele scores. Where we did find evidence for reverse causation, this was more frequent among physical health and educational outcomes than mental health outcomes. The lack of association could be due to a genuine causal effect of alcohol on mental health or confounding in the observed association; however, it could also be due to increased measurement error in mental health outcomes (e.g. due to symptom heterogeneity). Additionally, GWAS sample sizes are generally smaller for mental health outcomes and as a result there have been fewer genetic variants identified for these phenotypes meaning that instruments for mental health are not as strong as for phenotypes such as BMI and education. Several of the mental health phenotypes are also subject to selection, e.g. the rarer disorders such as schizophrenia, ASD and bipolar disorder. The onset of these are all likely to have occurred before the age of entering UKB; however, the percentage of participants reporting these outcomes is much smaller than expected in the general population. This is consistent with the levels of non-participation for extreme phenotypic manifestations (e.g. a diagnosis of schizophrenia) that have been observed in previous studies. We have used allele scores to investigate the whole range of phenotypic outcomes across these levels of genetic liability but these scores are also related to levels of participation in studies, although to a lower degree.^26–28^

It is important to note that although MR does provide protection from reverse causation (and recent methods such as MR Steiger aim to investigate this^29^) these analyses are not ‘immune’ to bias from reverse causation, as previously discussed.^4^^,^^30^ Reverse causality can also arise from the processes leading to the outcome of interest, rather than simply the outcome itself.^31^ A particular issue to be wary of when using MR to investigate reverse causation is misspecification of the primary phenotype. Without a good understanding of the underlying biology of the genetic variants included within a genetic instrument, it can be difficult to determine the direction of causation.^4^ This is because genetic variants could have their primary influence on either the exposure or outcome of interest, which is a major focus of the recently developed GRAPPLE (Genome-wide mR Analysis under Pervasive PLEiotropy) method.^32^

### Strengths and limitations

There are a number of limitations to consider in this study. First, UKB had a response rate of ∼5% and is widely acknowledged to not be representative of the general population of the UK.^33^ For example, UKB contains a lower proportion of current smokers and has lower rates of 5-year mortality than the UK population. Given that agreeing to take part in UKB is associated with a number of characteristics that are likely to reflect health and social status, then it is possible that in this study (which aims to investigate the bias due to confounding and reverse causation in observational studies), the effects of such harmful behaviours may be limited. However, as a preliminary check, we tested the association between depression and alcohol consumption to ensure the association was as we would expect in the general population. Second, we used instruments based on the genome-wide significance threshold of *P* < 5 × 10^–8^ in order to reduce potential for pleiotropy. A more stringent *P*-value threshold ensures that variants are robustly related to the phenotype of interest, reducing noise in the instrument. However, in some cases this results in a limited number of variants included within our instruments, which is likely to impact on the strength of these instruments, limiting the power of our analyses to identify effects. Third, we were restricted to the use of GWASs that did not include UKB as part of the discovery sample. This limits the number of available GWASs from which to select instruments and again may impact on the strength of our instruments as the smaller sample sizes are likely to result in the identification of fewer variants. However, using variants from more recent GWASs including UKB could lead to artificially inflated estimates as a result of overfitting the data. Fourth, if there is a causal relationship between an exposure (e.g. alcohol consumption) and an outcome (e.g. depression), then the SNPs contained within our allele scores for alcohol may also appear to act as instrumental variables (IVs) for depression. Methods such as MR Steiger,^29^ which can help to select valid IVs, should therefore be used to guard against this. Fifth, the interpretation of this approach becomes complicated when we are instrumenting time-varying phenotypes, such as frequency of consumption, which can both cause the outcome of interest but also be altered as a result of it. When considering time-varying phenotypes it is important to bear in mind certain nuances.^34^ For example, if we were to look at alcohol use and CVD, although the occurrence of a heart attack cannot influence pre-existing factors, it is possible that the disease process leading to the heart attack can. However, our approach uses genetic liability for these phenotypes, which is consistent across the life course. Supplementary Figure S1 (available as Supplementary data at *IJE* online) illustrates the potential relationship between depression and alcohol use across time. The directed acyclic graph (DAG) suggests that we can estimate the total effect of genetic liability for depression on drinking status at a particular time point, although this does not necessarily tell us about the influence of depression at a certain time point. Evidence of an observational association between alcohol consumption and depression, and also of a causal effect from depression to alcohol consumption may reflect a true bidirectional relationship. Where polygenic scores are available for both the exposure and the outcome, this can be formally tested. Sixth, as just mentioned, our approach uses genetic liability for an outcome (here, alcohol consumption) rather than the outcome itself. This means we are unable to estimate the effect size for the relationship we are investigating but does provide evidence of whether or not a causal effect exists.

## Conclusion

These analyses illustrate the potential for reverse causation to distort the results of conventional observational studies. Here, our findings suggest that observed associations suggesting beneficial effects of alcohol consumption may be largely due to confounding by reverse causation and provides some evidence for the sick quitter hypothesis. Our analyses present a useful proof of principle that illustrates how reverse MR could be routinely used to directly test reverse causation in observational epidemiological studies where bidirectional MR is not possible. It may also be possible to use this approach to adjust conventional observational estimates for reverse causation.

## Ethics approval

UK Biobank is approved by the National Health Service National Research Ethics Service (ref 11/NW/0382; UK Biobank application number 9142).

## Supplementary Material

dyac165_Supplementary_Data

## Data Availability

The UK Biobank data set used to conduct the research in this paper is available via application directly to UK Biobank. Applications are assessed for meeting the required criteria for access, including legal and ethics standards. More information regarding data access can be found at http://www.ukbiobank.ac.uk/scientists-3/.
