# Supplementary material for: Using allele scores to identify confounding by reverse causation: studies of alcohol consumption as an exemplar
Source: Int J Epidemiol. 2022 Aug 18;52(2):536–44. doi: 10.1093/ije/dyac165 (PMC10114122; doi:10.1093/ije/dyac165)
Supplement: dyac165_Supplementary_Data [file dyac165_supplementary_data.zip › dyac165_Supplementary_Data/ije-2021-09-1400-File010.docx]

**Using allele scores to identify confounding by reverse causation: studies of alcohol consumption as an exemplar**

**Sallis, H.M., et al.**

**Supplementary Materials**

# **Alcohol intake – deriving weekly consumption**

Self-reported alcohol consumption was measured at the baseline assessment visit and two follow up clinics. Participants were asked about 1) current drinking status (‘never’, ‘current’ or ‘former’), 2) frequency of drinking (‘daily or almost daily’, ‘three or four times a week’, once or twice a week’, ‘one to three times a month’, ‘special occasions only’, ‘never’), and 3) average weekly or monthly intake of certain types of alcohol (‘beer and cider’, ‘champagne and white wine’, ‘fortified wine’, ‘red wine’, ‘spirits’, ‘other’).

The following units were used to estimate weekly consumption: 1) pint of beer/cider: 2.3 units, 2) glass of wine: 1.6 units, 3) spirits: 1 unit, 4) fortified wine: 1 unit, 5) other: 1.5 units. For participants reporting monthly drinking (rather than weekly), the number of units consumed per month were divided by four to give an equivalent value of weekly units.

# **Quality control of UK Biobank genetic data**

Quality Control filtering of the UK Biobank data was conducted by R.Mitchell, G.Hemani, T.Dudding, L.Corbin, S.Harrison, L.Paternoster (1) as described below and in the published protocol (doi:10.5523/bris.1ovaau5sxunp2cv8rcy88688v).

# **Genotyping and imputation**

The full data release contains the cohort of successfully genotyped samples (n=488,377). 49,979 individuals were genotyped using the UK BiLEVE array and 438,398 using the UK Biobank axiom array. Pre-imputation QC, phasing and imputation are described elsewhere (2). In brief, prior to phasing, multiallelic SNPs or those with MAF ≤1% were removed. Phasing of genotype data was performed using a modified version of the SHAPEIT2 algorithm (3). Genotype imputation to a reference set combining the UK10K haplotype and HRC reference panels (4) was performed using IMPUTE2 algorithms (5). The analyses presented here were restricted to autosomal variants using a graded filtering with varying imputation quality for different allele frequency ranges. Therefore, rarer genetic variants are required to have a higher imputation INFO score (Info>0.3 for MAF >3%; Info>0.6 for MAF 1-3%; Info>0.8 for MAF 0.5-1%; Info>0.9 for MAF 0.1-0.5%) with MAF and Info scores having been recalculated on an in-house derived ‘European’ subset.

# **Data quality control**

Individuals with sex-mismatch (derived by comparing genetic sex and reported sex) or individuals with sex chromosome aneuploidy were excluded from the analysis (n=814).

# **Ancestry**

We restricted the sample to individuals of white British ancestry who self-report as “White British” and who have very similar ancestral backgrounds according to the PCA (n=409,703), as described by Bycroft and colleagues (2).

**Degree of relatedness**

Estimated kinship coefficients using the KING toolset (6) identified 107,162 pairs of related individuals (2). An inhouse algorithm was then applied to this list and preferentially removed the individuals related to the greatest number of other individuals until no related pairs remain. These individuals were excluded (n=79,448). Additionally 2 individuals were removed due to them relating to a very large number (>200) of individuals.

**Summary**

In total, there were 151,301 individuals excluded from the genetic dataset on the basis of ethnicity, relatedness and other recommended exclusions (as described by Mitchell and colleagues (1)).

# **References**

1. Mitchell R, Hemani G, Dudding T, Corbin L, Harrison S, Paternoster L. UK Biobank Genetic Data: MRC-IEU Quality Control, version 2. Bristol, University of Bristol, 2019; https://doi.org/10.5523/bris.1ovaau5sxunp2cv8rcy88688v. (20 July 2022, date last accessed).

2. Bycroft C, Freeman C, Petkova D, Band G, Elliott LT, Sharp K, et al. The UK Biobank resource with deep phenotyping and genomic data. Nature. 2018;562(7726):203–9.

3. O’Connell J, Sharp K, Shrine N, Wain L, Hall I, Tobin M, et al. Haplotype estimation for biobank-scale data sets. Nat Genet. 2016;48(7):817–20.

4. Huang J, Howie B, McCarthy S, Memari Y, Walter K, Min JL, et al. Improved imputation of low-frequency and rare variants using the UK10K haplotype reference panel. Nat Commun. 2015;6(1):8111.

5. Howie B, Marchini J, Stephens M. Genotype imputation with thousands of genomes. G3 (Bethesda). 2011;1(6):457–70.

6. Manichaikul A, Mychaleckyj JC, Rich SS, Daly K, Sale M, Chen WM. Robust relationship inference in genome-wide association studies. Bioinformatics. 2010;26(22):2867–73.

**Supplementary Figures**

**
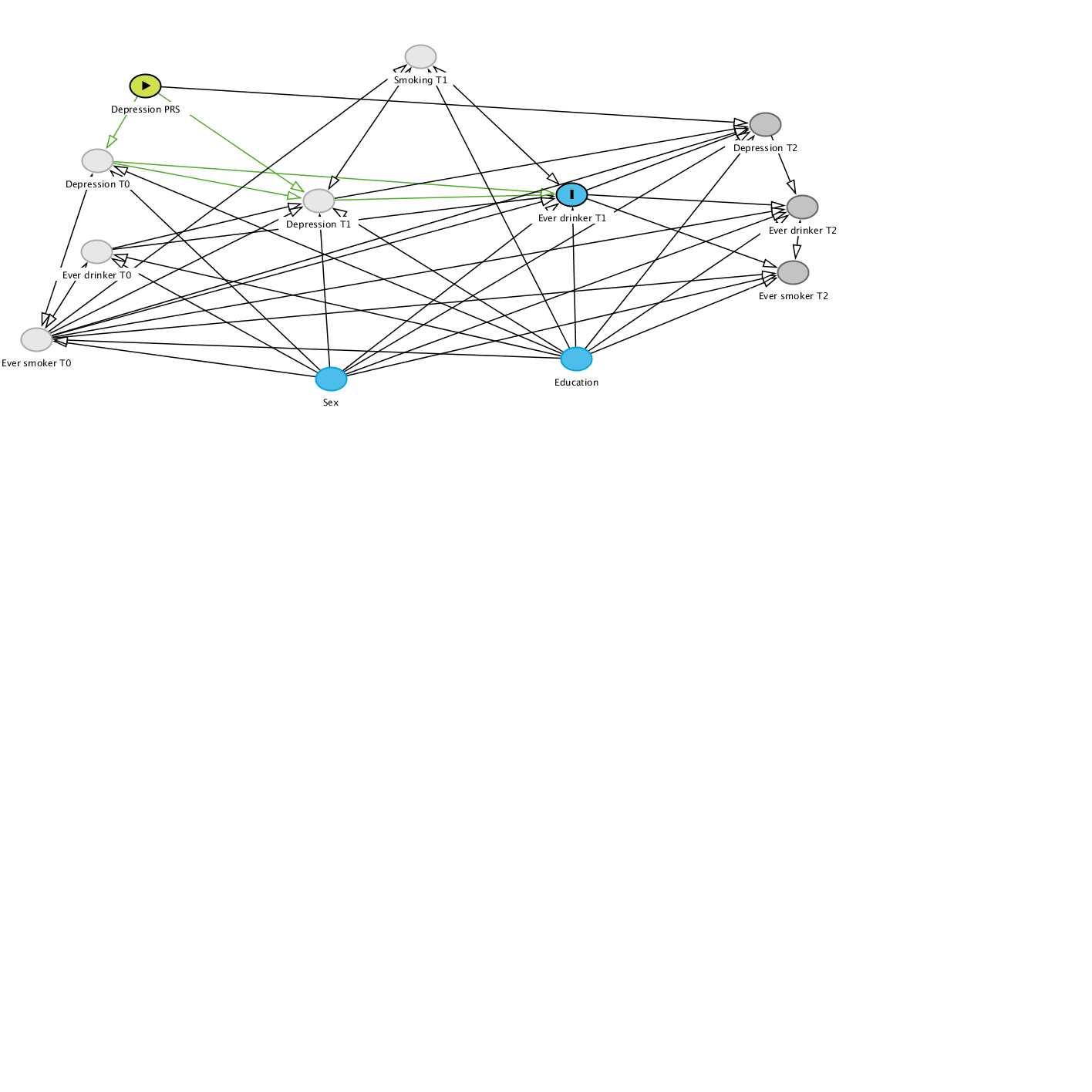
**

Figure S1. Directed acyclic graph of relationship between genetic liability for depression (depression allele score) and drinking status at time 1
